# Hemp Seed Cake Flour as a Source of Proteins, Minerals and Polyphenols and Its Impact on the Nutritional, Sensorial and Technological Quality of Bread

**DOI:** 10.3390/foods12234327

**Published:** 2023-11-29

**Authors:** Tatiana Capcanari, Eugenia Covaliov, Cătălina Negoița, Rodica Siminiuc, Aurica Chirsanova, Vladislav Reșitca, Dinu Țurcanu

**Affiliations:** Faculty of Food Technology, Technical University of Moldova, 9/9 Studentilor St., MD-2045 Chisinau, Moldova; eugenia.covaliov@toap.utm.md (E.C.); catalina.cerchez@toap.utm.md (C.N.); rodica.siminiuc@adm.utm.md (R.S.); aurica.chirsanova@toap.utm.md (A.C.); vladislav.resitca@adm.utm.md (V.R.); dinu.turcanu@adm.utm.md (D.Ț.)

**Keywords:** *Cannabis sativa* L., hemp seed cake flour, bread, protein, amino acids, phenols, antioxidant activity, sensorial acceptance, CATA

## Abstract

Hemp (*Cannabis sativa* L.) seeds contain a high concentration of proteins and biologically active compounds. The protein content is even higher in case of lipid part removal in oil production. The remaining part is considered a leftover, usually being used in animal feed. The aim of this study was to investigate the physicochemical composition of hemp seed cake flour, its nutritional quality and its impact on bread quality parameters. The properties of hemp seed cake flour were assessed in terms of protein quality, mineral composition, polyphenols and antioxidant activity. Hemp seed cake proved to be an important source of high-quality protein (31.62% d.m.) with the presence of eight essential amino acids. The biologically active potential of hemp seed cake has been demonstrated by the high content of polyphenols, especially those from the Cannabisin group. Hemp seed cake flour was incorporated in wheat flour at levels from 5 to 40% (*w*/*w*) to investigate its influence on bread quality parameters. The addition of hemp seed cake flour increased the total phenol content of bread, thus greatly enhancing the antioxidant activity. The protein content of bread was found to be enhanced from 11.11% d.m (control sample) to 18.18% d.m (for sample with 40% hemp seed cake flour). On the other hand, the addition of hemp seed cake flour led to decreased bread porosity, increased hardness and decreased resilience in the seed cake. Although, all bread samples recorded sensorial attributes ranging between “slightly like” and “like it very much”.

## 1. Introduction

Food has always been a basic necessity, but it is also often a pleasure that prompts people to buy more and more products, whether or not it is necessary [1,2]. In a world where food waste is becoming a “normality”, the problem of the resulting waste is increasingly pronounced, and the consequences for the environment are not at all negligible [3,4,5]. Food waste is among the main sources of environmental pollution and can also be an ethical issue in terms of global hunger [6,7,8,9]. For these reasons, correct food waste management must be a significant objective for consumers and companies operating in this industry [10,11,12].

Hemp (*Cannabis sativa* L.) is a low-cost, unconventional feed resource with a unique phytochemical composition and various uses (pharmaceutical industry, food industry, etc.) [13,14,15]. However, it has long been controversial because of the confusion about the health risk due to the increased content of hallucinogenic substances (tetrahydrocannabiol, THC) [16,17]. Taking as a starting point the multiple therapeutic effects ascribed to the content in active biocompounds and the content of THC below the toxicity limits (≥0.2%), the use of industrial (*Cannabis sativa* L.) hemp has been rethought and acquired new valences [18,19,20,21]. More than that, starting in January 2023, the new Common Agricultural Policy (CAP), adopted by European Council and the European Parliament, entered into force. The new CAP stipulated that the permitted THC level in hemp products was raised from 0.2% to 0.3% [22,23]. Hemp seeds stand out due to their fairly high protein content (22.17%) with high biological value, reflected in a high essential amino acid content [24,25,26]. Equally, hemp seeds have a high energy value due to their fat content (26.25% (*w*/*w*) to 37.50%), to which the beneficial fatty acid structure is added. Nowadays, the by-products or leftovers of hemp seeds (cakes from oil factories) are unutilized for human consumption, being used in animal feed [27,28,29]. However, hemp seed cake contains up to 50% proteic substances, 9–20% lipids, 6–7% dietary fibre, important amounts of minerals and could be successfully used in the manufacture of food products for human consumption [30,31,32,33,34].

Bread has been an important staple food product to many cultures over the centuries [35,36]. It is referred to as the “staff of life” in the *Bible* and it is still the most eaten product in some regions [37]. On the other hand, several studies have found a significant relation between bread consumption and body weight, abdominal fat distribution, high postprandial glucose, etc. [38,39,40,41]. This may be attributed to the fact that nowadays most bread is refined, with a low content of fibre and vitamins, thus providing quick and easily digestible carbohydrates. Thus, people are encouraged to restrict bread from their diet or to give preference to breads enriched with fibres, proteins and other biologically active compounds [41,42,43]. As a result of these considerations, consumer demands have increased; they want their bakery products to have a satisfying taste and texture, while respecting nutritional health trends [42,44]. To satisfy all these attributes, in the challenging global context, the bakery industry has to face many challenges [45,46]. Thus, innovative solutions are sought to be able to provide distribution networks with tasty and nutritious bakery products, which keep their freshness longer and which are obtained using sustainable means [47,48,49,50].

Therefore, the objective of this study was to evaluate the nutritional and biological value of hemp (*Cannabis sativa* L.) seed cake and therefore the impact of the wheat flour substitution by hemp seed cake on the sensory, physicochemical, textural and technological characteristics of bread.

## 2. Materials and Methods

### 2.1. Materials

Bread formulations used premium-quality wheat flour, 550 type, which was purchased from the local market; hemp (*Cannabis sativa* L.) cake, a by-product remaining after hemp seed cold pressing, which was offered by a local enterprise “Mira OSF” SRL; drinkable water; salt; and leaven.

The hemp seeds used for oil production were grown on the territory of the Republic of Moldova, in the 2022 harvesting year. Hemp seed cake was ground using a Kenwood cooking chef XL (Kenwood, Hampshire, UK) mill and manually sifted through a sieve with 200 μm cells. In order to obtain the flour mixes, the hemp seed cake flour was mixed with wheat flour in different hemp/wheat flour ratios (0/100, 5/95, 10/90, 15/85, 20/80, 30/70 and 40/60).

The leaven used in bread making was a microbial consortium, which was obtained through spontaneous fermentation of a mixture of wheat flour and water (0.75:1), assured mainly by spontaneously growing yeasts and lactic acid bacteria (LAB). The choice of spontaneous fermentation was justified by the proven positive effects of the native microbiota on bread quality. According to Ramos et al. 2021, the spontaneous leaven contains an optimal ratio of LAB and yeast 100:1 [51]. In the same context, Di Cagno et al. 2002 mentioned that leaven lactobacilli positively influenced the softening of the dough during fermentation in comparison with the chemically acidified dough [52]. Similarly, Siminiuc and Turcanu 2020 demonstrated the positive effect of spontaneous sourdough on the porosity of the final product against the product obtained by using commercial yeast [53]. Several studies proved the benefits of leaven microbiota on proteolytic processes that enhance the bread’s flavour, amylolytic and phytase activity, volatile and antimicrobial production, capacity of expansion and gas retention, and increased shelf life [54,55,56].

In order to be used, a fresh leaven must be refreshed with flour and water, a process also called backslopping, which could be an obstacle to the widespread use of the dough [57]. Thus, to obtain the leaven, systematic refreshments (feeding) with flour and water were conducted over 30 days at 22–26 °C. After 30 days from the moment of the last refreshment, 12 h of fermentation at 22–26 °C is needed in order to proceed to the technological process of bread dough manufacturing. During these 12 h, the microorganisms present in the environment grow, producing new substances that affect the rheological and organoleptic properties of the dough. To confirm the leaven quality and its possibility of being used as a dough starter, total titratable acidity (TTA) and pH were measured. After 12 h of fermentation, the optimal values of TTA (6.87 degrees acidity) and pH (4.2) necessary for the dough-forming process were reached. These values were also confirmed by previous studies [53,58]. The leaven quality was appreciated and sensorial. The sweet and sour fermented milk flavour was also an indicator that denotes the leaven’s readiness.

### 2.2. Methods

Properties such as the acidity, moisture, ash and wet gluten content of the premium-quality wheat flour and bread were determined according to standardized methods provided in the international methods of the AACC (02–31.01 for acidity, 44–15.02 for moisture content, 08–16.01 for ash content and 38–10.01 for wet gluten) [59] and compared to values stipulated in national regulations [60].

The proximate analysis of hemp seed cake flour was conducted according to the standard AOAC method [61]. Moisture (AOAC 930.15), crude fat (AOAC 2003.05), crude fibre (AOAC 978.10) and ash (AOAC 942.05) were analysed. The crude protein content was expressed as the sum of proteinogenic amino acids. All measurements were conducted in triplicate.

### 2.3. Protein Quality Determination

#### 2.3.1. The Analysis of Amino Acid Composition in Hemp Seed Cake Protein

The content of constituent amino acids of hemp seed cake flour was determined according to Dudok et al. (1997) [62]. The method is based on the hydrolysis of proteins into constituent amino acids under the action of acids (HCl (6N), 105 °C, 24 h) followed by their identification with the AAA 339 “Mikrotechna” analyser (Mikrotechna Praha, Prague, Czech Republic).

#### 2.3.2. Scoring of Amino Acids

The amino acid score (*AAS*) was calculated for adults using the standard method according to FAO with the formula [63]:

(1) $$AAS\;=\;\frac{essential\;AA\;content\;in\;Hemp\;cake\left(wheat\right)\;protein,\%}{recommended\;essential\;amino\;acids,\%}$$

#### 2.3.3. Protein Efficiency Ratio

The Protein Efficiency Ratio (PER) was calculated taking into account the amino acid content of the protein of hemp seed cake flour, mainly Leucine (Leu), Proline (Pro), Tyrosine (Tyr), Methionine (Met) and Histidine (His). Certain combinations of the mentioned amino acids can enhance or diminish the growing ability of animals. The calculations were performed according to Kowalczewski et al. (2019) [64] using Equations (2)–(4).

PER1 = −0.684 + 0.456 × Leu − 0.047 × Pro,(2)

PER2 = −0.468 + 0.454 × Leu − 0.105 × Tyr,(3)

PER3 = −1.816 + 0.435 × Met + 0.78 × Leu + 0.211 × His − 0.944 × Tyr,(4)

where Leu, Pro, Tyr, Met and His are the amount of the amino acid in g/16 g N or g/100 g protein.

### 2.4. The Analysis of Mineral Composition of Hemp Seed Cake Flour

The content of the minerals Mg, K, P, Mn, Fe, Zn and Cu in hemp seed cake flour was determined using AOAC methods as described by James [65]. The percent of the population reference intake (PRI) of minerals was calculated according to the European Food Safety Authority (EFSA) recommendations. The PRI values were 350 mg for Mg, 3500 mg for K, 550 mg for P, 3 mg for Mn, 11 mg for Fe, 14 mg for Zn and 1.6 mg for Cu [66].

### 2.5. The Analysis of Polyphenols in Hemp Seeds and Hemp Seed Cake Flour by HPLC

For polyphenols’ identification, the Agilent 1200 HPLC system equipped with quaternary pump, solvent degasser, autosampler and UV-Vis detector with a photodiode (DAD) coupled with a mass detector (MS) single quadrupole Agilent model 6110 (Agilent Technologies, Santa Clara, CA, USA) was used.

The separation of the compounds was carried out on a Kinetex XB C18 column, dimensions 4.6 × 150 mm, with 5 μm particles (Phenomenex, Torrance, CA, USA), using the mobile phases (A) water + 0.1% acetic acid and (B) acetonitrile + 0.1% acetic acid, for 30 min, at a temperature of 25 °C, with a flow rate of 0.5 mL/min and with the following gradient (expressed in % B): 0 min, 5% B; 0–2 min, 5% B; 2–18 min, 5–40% B; 18–20 min, 40–90% B; 20–24 min, 90% B; 24–25 min, 90–5% B; 25–30 min, 5% B.

**Figure 1:**
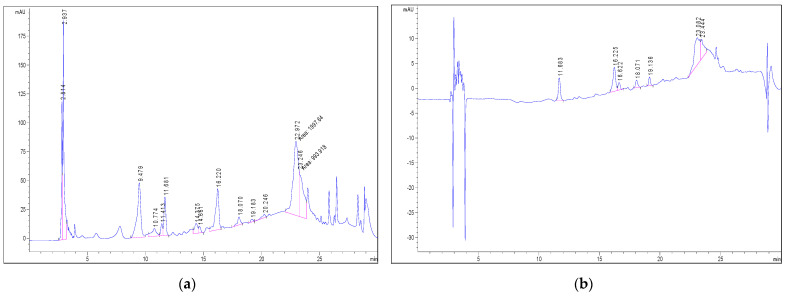
Peaks marked with retention time. The result of GC-MS analysis of the hemp seeds, the main components are shown as marked peaks with retention times ((**a**) λ = 280 nm, (**b**) λ = 350 nm).

The spectral values were recorded in the 200–600 nm range for all peaks. The chromatograms (Figure 1) were recorded at the wavelength λ = 280 and 350 nm.

For MS, the full-scan ESI positive ionization mode was used in the following working conditions: capillary voltage 3000 V, temperature 350 °C, nitrogen flow 7 L/min, collision energy 100 eV and *m*/*z* 120–1200. Data acquisition and interpretation of results were conducted using Agilent ChemStation software, version B.02.01 SR2.

The phenolic compounds were identified by comparing the retention time, UV-Vis absorption and mass spectra with those of the standard compounds and with the data from the specialized literature.

### 2.6. Bread-Making Procedure

Bread dough was prepared by mixing the wheat flour and/or hemp cake/wheat flour mixtures with tap water and leaven. The optimum content of the used leaven was determined experimentally. Smaller and higher leaven amounts (compared to 675 g leaven for 1200 g of wheat flour) resulted in bread with porosity and aspect shortcomings. When using a higher amount of leaven, the bread taste was sour.

The obtained mass was gently mixed for 5 min using a KitchenAid (Artisan^®^ Series 5 Quart Tilt-Head; Greenville, OH, USA) dough mixer (tilt-head stand mixer) and left for autolysis for 30 min. Then, the salt was added and the dough was kneaded for 10 min until an optimal consistency was achieved. The dough was transferred into a bowl, covered with plastic, and left to ferment at 30 °C and a relative humidity of 80% for 60 min, after which it was placed on the moistened worktable, spread out in a thin layer, folded into 4 and left for a repeated rest. The stretching and folding operation was repeated 3 times at an interval of one hour. Afterward, the obtained dough was divided into 620 g pieces, shaped, put in the fermenting mould and put in the refrigerator for cold fermentation at 4 °C for 12 h. After cold fermentation, the dough was taken out from the moulds, put on the backing sheet and at the surface of each piece, light oblique notches were made with the tip of a knife. The dough pieces were baked in a preheated oven (Fimar, Via Del Tesoro, 301 Villa Veruccihio, Rimini, Italy) at +227 °C for 45 min. The baked bread was cooled at room temperature (+21 °C) for 2 h before its properties were assessed. Bread formulations are presented in Table 1, where the bread with a 0% replacement of wheat flour was considered the control sample.

To ensure the applicability of the technological process described in the paper, all bread samples were prepared in an artisanal bakery, which simulates semi-industrial conditions.

### 2.7. Determination of Baking Loss

Baking loss was determined by weighing the dough before and after baking according to the formula:

(5) $$BL\%=\frac{m_{dough}-m_{bread}}{m_{dough}}\times 100\%,$$

where *BL* is the baking loss value, %;

*m_dough_* is the mass of the dough, g;

*m_bread_* is the mass of the baked bread.

### 2.8. Determination of Porosity

Porosity was determined as described by Rumeus and Turtoi (2013) using the Juravliov device [67].

### 2.9. Determination of Volume of Bread

The volume of the bread samples was determined according to Lu et al. (2010), by the rapeseed displacement method [68].

### 2.10. Determination of Water Activity

Water activity was determined according to Hussain et al. (2021) using the Rotronic water activity meter (INSTRUMART, Williston, VT, USA) [69].

### 2.11. Analysis of Bread Crumb Texture

Bread crumb texture analysis was performed after cooling the bread for 8 h. Small pieces (3 × 3 × 3 cm) were cut from the middle of each bread. The texture profile measurements were taken using a TA.HDplusC texture analyser (Stable Micro Systems, Godalming, UK) with a 36 mm diameter cylindrical probe, 50% compression and a test speed of 1.0 mm s^−1^, according to Young (2012) [70].

### 2.12. Evaluation of Colour Parameters

The colour evaluation of bread was performed using the Konica Minolta colourimeter CR-400 (Osaka, Japan). Chromatic parameters *L*, *a* and *b* were measured for each bread sample. To assess the impact of hemp seed cake flour addition, the colour difference Δ*E* and whiteness index (*WI*) were determined using Equations (6) and (7).

(6) $$∆E=\sqrt{{\left(L_{sample}-L_{0}\right)}^{2}+{\left(a_{sample}-a_{0}\right)}^{2}+{\left(b_{sample}-b_{0}\right)}^{2}},$$

(7) $$WI=100-\sqrt{{\left(100-L\right)}^{2}+a^{2}+b^{2}}$$

### 2.13. Total Polyphenol Content (TPC)

The total polyphenol content in bread samples was determined by the Folin–Ciocalteu method described by Makkar et al. (2003) [71].

### 2.14. Antioxidant Activity (AA)

Analysis of the free radical scavenging ability against the DPPH• free radical was performed according to Lin and Zhou (2018) [72]. Results were expressed as mg of Trolox per kg of product.

### 2.15. Consumer Test and Sensory Analysis

The consumer acceptance test was performed within the Faculty of Food Technology, Technical University of Moldova, with 73 consumers (staff and students, 54.8% females and 45.2% males), aged between 18 and 62 years old. The consumers were offered a tasting sheet, in which the main quality parameters of the bread and its grading scale were indicated. In order to be evaluated, bread samples were cut into 25 mm edge cubes [73], similar to Elía’s [74] study, with a 20 mm cube edge.

The assessed quality parameters were overall appearance, colour, texture, odour, and taste. The quality of bread was evaluated using a 9-point hedonic scale from “dislike extremely” to “like extremely”, ranging from 0 to 9 points, respectively (1—dislike extremely, 2—dislike very much, 3—dislike, 4—dislike slightly, 5—neither like nor dislike, 6—like slightly, 7—like, 8—like very much and 9—like extremely).

### 2.16. Check-All-That-Apply (CATA)

A check-all-that-apply questionnaire was performed according to Biró et al. (2020). As part of the experiment, assessors were asked to identify which sensory attribute was present in the sample. A product’s attributes are usually based on its sensory characteristics, but can also include hedonic terms, emotions, and non-sensory qualities. According to the method, there was no limitation on the number of terms that could be selected by the assessors [75]. Based on consensus, 12 trained assessors compiled a list of terms to be used. The terms are listed in Table 2.

### 2.17. Statistical Analysis

Significant differences between the samples were analysed using analysis of variance (ANOVA) at a 5% level of significance. CATA analysis was performed using XLStat Software, version 7.5.2, for Excel. All the experiments were carried out in three replicates, and data were reported as the mean ± standard deviation (SD).

## 3. Results

### 3.1. Characteristics of Wheat Flour and Hemp Seed Cake Flour

The quality of the used flours was evaluated in terms of moisture, ash, acidity and wet gluten content for wheat flour, whereas for hemp seed cake flour, in addition to moisture and ash content, quality was expressed in terms of total fat and protein content, amino acid composition, minerals and phenols. The values of the above-mentioned quality indicators are presented in Table 3.

The values of the determined parameters for the wheat flour correspond to the national legislation [60]. It is known that the main compounds of premium-quality wheat flour are starch and proteins, thus it is lacking any biologically active compounds or complete proteins. In this regard, many studies have been conducted in order to improve bread’s nutritional quality [64,76,77].

According to the obtained values for the hemp seed cake flour, it can be stated that this seed cake product may be considered a rich protein and fibre source with broad prospects for use in supplementing food products. The crude protein content of hemp seed cake flour was 31.62% d.m. Similar values were reported previously by Rajasekhar et al. (2021). Values of 32.06% and 31.22% for the protein content of hemp seed cake were also reported by Mierliță (2019) [78,79]. According to Xu et al. (2022), hemp protein has a better digestibility (88–91%) than soybean protein (71%) that is widely used in food products [80]. The same is stated by Aluko (2017), who’s study mentioned that hemp proteins have a higher bioavailability for the human body due to the fact that about 70% of them are soluble at pH 4.00–6.00 [81], while most plant proteins are insoluble at such pH conditions [82]. Thus, supplementing the bread with hemp seed cake flour will enhance its protein content. At the same time, it is known that hemp proteins contain essential amino acids that, in combination with wheat flour amino acids, could increase their total bioavailability [83,84].

In this study, the protein quality of the local hemp seed cake flour was evaluated by assessing the amino acid composition (Table 4). In order to prove the hemp seed cake flour’s protein superiority over wheat flour protein, the amino acid score (AAS, %) was calculated (Table 4).

The obtained data revealed the presence of eight essential amino acids (threonine, valine, methionine, isoleucine, leucine, phenylalanine, lysine and histidine), one semi-essential amino acid (arginine), nine non-essential amino acids (aspartic acid, serine, glutamic acid, proline, glycine, alanine, cysteine and tyrosine) and one non-protein amino acid (γ-aminobutyric acid (GABA)). House et al. (2010) reported the presence of the same amino acids in the hemp seed meal, but the amount of each amino acid was significantly different from our results [86]. This fact might be explained by the crude fat content of hemp seed cake flour that was found to be higher (10.2% d.m.) than that in our study (8.19% d.m.).

The share of essential amino acids accounted for 37.13%, semi-essential acid arginine reached a value of 10.93% and non-essential amino acids amounted to 51.94%. It is known that the essential amino acid (E) ratio in the total amino acid content (T) is very important; hence, when they are in excess, they can be considered sources of non-essential amino acids. According to Heger (2003), the optimum ratio for well-balanced egg protein was found to be 0.4, whereas the value obtained in our study (0.37) was close to the one mentioned above [87]. The promising value of the E:T ratio in hemp seed cake flour (0.37) is also supported by another study by Heger et al. (1987) that mentions the maximum nitrogen retention being attained at an E:T ratio of 0.38 in rats fed on diets [88].

As shown by the data presented in Table 5, as well as many other studies [89,90,91,92], the limiting amino acid in wheat flour is lysine, with a score of 63.33%. At the same time, lysine is the second limiting amino acid in hemp seed cake flour (AAS—85.77%), after valine with a score of 74.10%. Although, compared to wheat flour, the lysine content in hemp seed cake flour higher. The analysis of the chemical score for the hemp seed cake flour amino acids suggests their possible combination with wheat proteins, a fact confirmed by several studies [30,93]; that is, the hemp seed cake flour proteins are rich in lysine and contain less valine than wheat flour proteins. Thus, by combining these flours, an equilibrium among amino acids can be attained.

The protein quality of the hemp seed cake flour was also assessed by determining the PER, an indicator that represents a measure that evaluates the ability of a protein to support growth in animals. The values of PER are presented in Table 5.

In this context, a value of 2 or higher is often used as a benchmark for protein sources that are considered to be of higher quality. This means that the protein source has a good amino acid profile and can effectively support growth and development [94,95,96]. In this respect, many studies were dedicated to the effect of hemp seeds or seed cakes on animal nutrition and growth [28,97,98]. Concerning protein quality, it is also worth mentioning the presence in hemp seed cake flour of GABA that is known for its potential health benefits. Nowadays, many efforts are being made to develop new technologies for GABA food enrichment [99].

Hemp seed cake flour was found to be a valuable source of most of the analysed macro- and microminerals (Table 6).

Taking into account the obtained values, it can be concluded that the consumption of 100 g hemp seed cake flour per day can cover 85–96% of the daily requirements for Zn and Fe. Minerals Mg, P, Mn and Cu were also highlighted with a high content, covering approximately 252.39, 336.62, 438.38 and 120.12% of the PRI, respectively. The comparison of obtained values with the same data for wheat flour [100] showed that the hemp seed cake flour is superior to wheat flour concerning the amount of all determined minerals.

Mourtzinos et al. (2018) proposed an extraction process to recover polyphenols resulting from hemp seed cake after the seed oil pressing and demonstrated that hemp solid waste is a rich source of functional constituents [101]. This research, therefore, aimed at determining and identifying the polyphenol composition of hemp seed cake flour (Table 7).

The estimated relative amounts of individual phenols were expressed as the individual peak area reported in contrast to the total peak area from chromatograms (Figure 1). The peaks marked with retention times are shown in Figure 1, and the detected components are shown in Table 8. According to the analysis of GC-MS, 14 phenolic components were detected in the hemp seed cake flour. The highest relative content was obtained for Cannabisin F (151.56 mg/kg hemp seed cake flour), with a share of 28.30% from the total phenol content. Apart from Cannabisin F, small amounts of Cannabisin A, B, C and Q were detected. Other phenolic compounds that prevail in hemp seed cake flour are Grossamide, 2-Hydroxybenzoic acid and N-trans-Coumaroyltyramine, with shares in the total polyphenol amount of 14.32, 11.13 and 10.52%, respectively.

These findings are completely consistent with numerous investigations that identified the specific abundance of cannabisins in hemp seeds [75,76]. Other studies reported the presence of some additional phenolic compounds in hemp seeds and cake, and this fact may be due to the different methods and solvents used in the research studies [102,103,104,105]. The use of mass spectrometry in analysing hemp seeds and seed cake flour phenols has expanded our understanding of their complex composition and potential health benefits. As research continues, we are likely to uncover even more insights into how these bioactive compounds can positively impact human health. Incorporating hemp seed cake flour into a balanced diet could offer a natural way to harness these potential benefits, contributing to overall well-being.

### 3.2. Impact of Hemp Seed Cake Flour on Bread Quality

#### 3.2.1. Impact of Hemp Seed Cake Flour on Bread Sensory Profile

##### Consumer Test and Sensory Analysis

**Figure 2:**
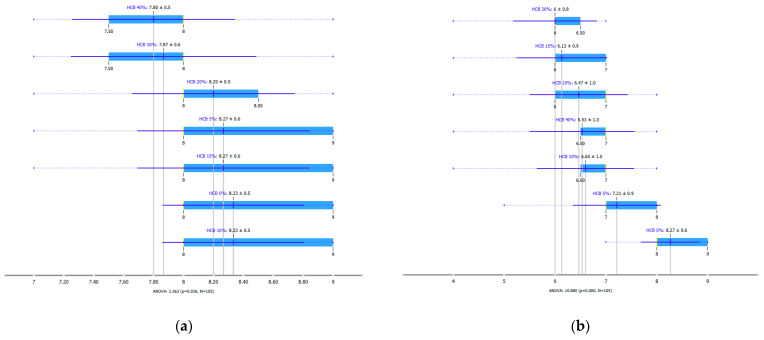

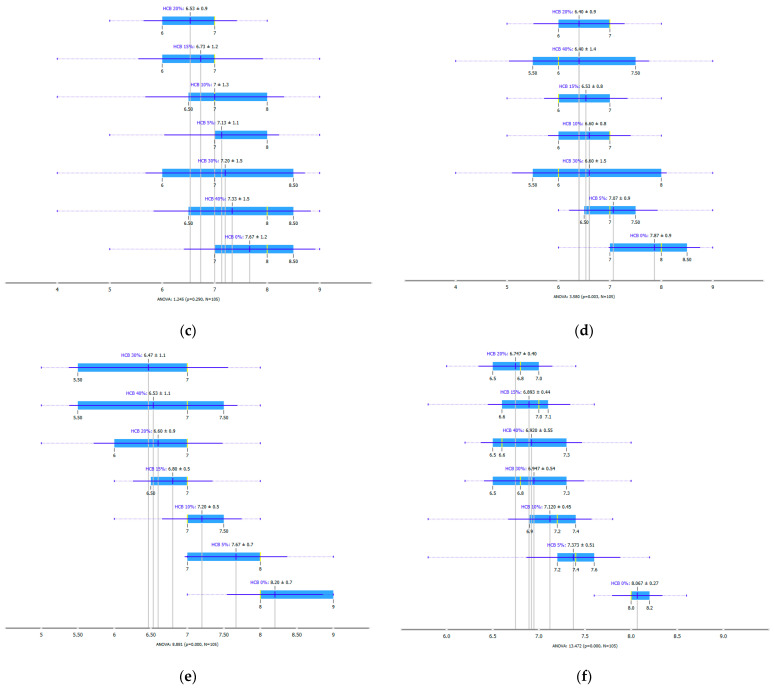
Boxplot for the consumer acceptance test: ((**a**). overall appearance, (**b**). crumb colour, (**c**). odour, (**d**): taste, (**e**). texture and (**f**) overall acceptance) using a nine-point hedonic scale. HCB0%—control sample, HCB5%—bread with 5% addition of hemp seed cake flour, HCB10%—bread with 10% addition of hemp seed cake flour, HCB15%—bread with 15% addition of hemp seed cake flour, HCB20%—bread with 20% addition of hemp seed cake flour, HCB30%—bread with 30% addition of hemp seed cake flour, HCB40%—bread with 40% addition of hemp seed cake flour.

In order to evaluate the impact of hemp seed cake flour on bread’s sensory profile, consumer tests and check-all-that-apply (CATA) analyses were performed. The results of sensory analysis are presented in the form of BoxPlots according to Jaimes (2015) [106]. Figure 2a–f allow for a general comparison of each bread sample with respect to each sensorial parameter. The tiny blue vertical line inside each box represents the obtained average. The box-plot analysis represents an attempt to identify the best and worst evaluated bread. It worth mentioning that in this analysis, the six descriptors (a. overall appearance, b. crumb colour, c. odour, d. taste, e. texture and f. overall acceptance) of the model are assumed to have equal weight.

The visualization of the overall appearance score (Figure 2a) proves that all samples have been appreciated using scores ranging from 7.80 (for HCB40%) to 8.33 (control and HCB10% sample). On the hedonic scale, the assigned values are situated between “like” and “like extremely”. Breads with a higher content of hemp seed cake flour (HCB30% and HCB40%) generated a higher variance in the attribute perception by the evaluators. This may be explained by the consumer preferences are that still inclined towards bread made from wheat flour. At the same time, the same source stated that the number of consumers that buy rye, bran or whole grain bread is increasing [107]. In the same context, Turcanu (2023) reveals that bread manufacturing trends and consumer preferences are leaning towards long-fermented products and artisanal technologies [108]. Nowadays, according to Nicolosi et al. (2023), Italian consumers have clear intentions to adopt sustainable inclinations in the purchase of bakery products. These intentions are the result of a difficult decision-making process encompassing a number of variables, even including worries about the environment, health, and climate change [109].

When evaluating the crumb colour attribute, there was a statistical difference in the assessment of the samples using the Friedman test (*p* ≤ 0.05). The most appreciated samples, except for the control, in terms of crumb colour were HCB5%, HCB30% and HCB40%. The consumers who preferred the colour of the HCB30% and HCB40% samples mentioned the similarity of these samples with rye bread available in commercial networks. Korus et al. (2017) mentions an increase in colour acceptability after the supplementation of bread with hemp flour [110]. The assessment of odour showed the following results: the control sample, HCB30% and HCB40% were appreciated with the highest mean scores (7.67 for control, 7.20 for HCB30% and 7.33 for HCB40%). Consumers mentioned an increasing intensity of the pleasant pine nut-like odour with the increasing proportion of hemp seed cake flour.

The taste scores (Figure 2d) copied the results of the odour in a very similar way. The results of the sensory evaluation of bread crumb texture (Figure 2e) ranged in their mean grades from 6.47 (HCB30%) to 8.20 (HCB0%), being similar (4.7 to 7.1) to those found in the study of García-Gomez et al. (2021) in which the sensory descriptive analysis and hedonic consumer test of Galician-type breads were performed [111]. The overall acceptability visualization (Figure 2f) indicates the breads’ liking score decreases with the increasing rate of hemp seed cake flour in their formulations. Thus, for breads HCB30% and HCB40%, the attribute most correlated with overall acceptance was odour (*r* = 0.83 and 0.79 *p* ≤ 0.05). Meanwhile, taste was the most correlated characteristic for the overall acceptance of breads HCB15% (*r* = 0.72, *p* ≤ 0.05), HB20% (*r* = 0.75, *p* ≤ 0.05) and HCB10% (*r* = 0.81, *p* ≤ 0.05). Thus, participants rated the palatability of the bread with hemp seed cake flour as acceptable and the principle of adding hemp seed cake flour appeared to be a feasible option from the sensory point of view. Taking into account all of the above-mentioned factors, it would be necessary to improve all of the sensory properties that caused the shortfalls in overall acceptance. However, all the samples achieved mean scores higher than 6.74, a value that allows us to state that such a bread, also considering its nutritional quality, can find its consumer niche in the market. The baked breads are shown in Table 8.

##### Check-All-That-Apply (CATA)

The CATA questionnaire consisted of 22 terms (Table 2). The six least used terms were golden colour, woody odour, earthy odour, pleasant odour, sweet taste and granular. Based on Cochran’s Q test, the characteristics that do not statistically separate the samples were filtered away to obtain more accurate findings. From now on, the analysis only includes the 16 key features that are left.

**Figure 3:**
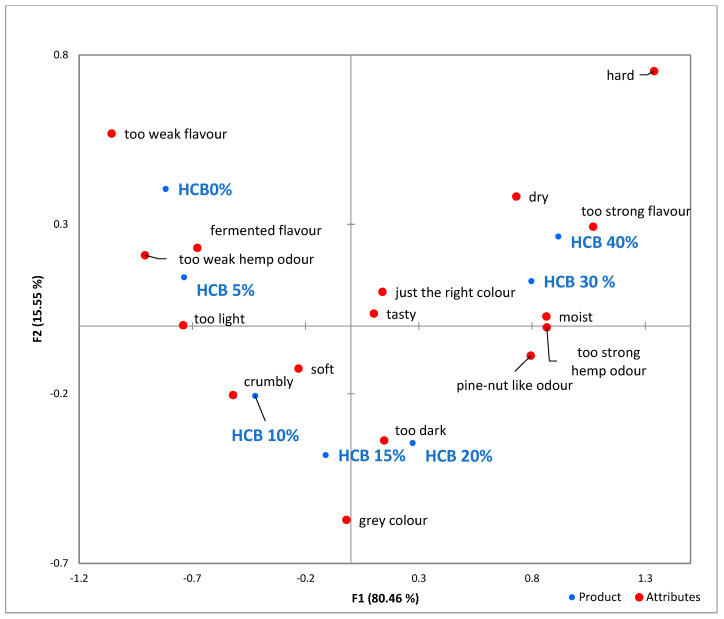
Visualized results of the check-all-that-apply (CATA) analysis of the seven bread samples. HCB0%—control sample, HCB5%—bread with 5% addition of hemp seed cake flour, HCB10%—bread with 10% addition of hemp seed cake flour, HCB15%—bread with 15% addition of hemp seed cake flour, HCB20%—bread with 20% addition of hemp seed cake flour, HCB30%—bread with 30% addition of hemp seed cake flour, HCB40%—bread with 40% addition of hemp seed cake flour.

Based on the correspondence analysis (Figure 3), each sample was associated with different attributes. The assessors noted that the flavour and hem odour of HCB0% and HCB5% samples were too weak. These samples exhibited characteristics such as a fermented flavour and were too light.

In this analysis, the most liked hemp-enriched breads were HCB30% and HCB40%. These results were reflected in the just the right colour, tasty and pine nut-like odour attributes, as well as the moist attribute. A grey and too dark colour beside crumbly texture appear in samples HCB10%, HCB15% and HCB20%. Negative attributes such as too strong hemp odour, too strong flavour, dry and hard were more associated with HCB20%, HCB30% and HCB40% samples.

#### 3.2.2. Impact of Hemp Seed Cake Flour on Bread Quality Characteristics

##### General Bread Quality Characteristics

In order to assess the impact of hemp seed cake flour addition on the main characteristics of bread, indicators such as baking loss, porosity, loaf volume, protein content, total polyphenol content and antioxidant activity were evaluated (Table 9).

Bread moisture content did not differ significantly (*p* ≤ 0.05), ranging within the limits of 45.43 to 47.27%. Baking loss of the samples was found between 9.16% and 12.73%. The control sample (HCB0%) significantly had the lowest baking loss, while HCB40% exhibited the highest baking loss.

The significant influence of hemp flour seed cake on bread volume and porosity was observed. The data (Table 10) show that in the case of breads with 30% and 40% hemp seed cake flour, loaf volume decreased to 1935.81 cm^3^ and 1846.62 cm^3^, respectively, from 2950.15 cm^3^ for control sample (HCB0%). The reduction in bread volume is consistent with the findings of Mikulek et al. (2019) who also worked with hemp flour and reported that hemp flour incorporation provided loaves with a lower volume [112]. Similar results were mentioned in numerous research studies aimed at enriching bread with protein or fibre sources from Germinated Chickpea Flour, apple pomace or wholegrain non-wheat flours [113,114,115]. The reduction in fibre- or protein-enriched bread volume is explained by different authors. Franco-Miranda et al. (2017) state that added proteins interfere in gluten formation and change the dough’s elasticity. Thus, the dough proves to be unable to retain the carbon dioxide resulting from the leavening process and a larger number of smaller alveoli in the crumb are created, in this way causing the volume and porosity decreases [116]. Besides this cause, Xu et al. (2021) mentioned the negatively affected hydration of gluten-forming proteins and gluten network formation due to competition for water between fibre and gluten protein, respectively [117].

The presence of hemp seed cake flour at different levels did not significantly (*p* ≤ 0.05) modify the breads’ acidity and water activity. On the other hand, the presence of different concentrations of hemp seed cake flour increased (*p* ≤ 0.05) the TPC of bread (182.35 ± 2.05, 216.17 ± 1.75, 225.84 ± 3.14, 256.43 ± 4.42, 308.97 ± 3.78 and 354.85 ± 2.87 mg GAE/kg for HCB5%, HCB10%, HCB15%, HCB20%, HCB30% and HCB40%, respectively) compared to control bread (HCB0%; 148.25 ± 3.24 mg GAE/kg).

Regarding antioxidant activity, there were significant differences (*p* ≤ 0.05) in antioxidant activity among bread samples with different concentrations of hemp seed cake flour. The highest antioxidant activity was found in breads with the addition of 40% of hemp seed cake flour: more than 2.75 times as compared to the reference wheat bread.

The positive effect of the incorporation of hemp seed cake flour on the crude protein content (PC) of fortified bread has been reported in numerous studies. Mikulek et al. (2019) outlined a PC of 19.29% d.m. (compared to 11.02 for the control counterpart) when fortifying the bread with partially defatted hemp flour (50% wheat flour substitution) [112]. Rusu et al. (2021) mentioned the same effect of hemp flour in the case of bread fortification [118]. Concerning hemp cake, Teterycz, et al. (2021) stated that this product is a valuable ingredient for developing bread with high nutritional value that provides an elevated intake of important nutrients such as proteins and macro- and microelements, especially iron, reporting a PC of 16.14% d.m. for the pasta fortified with hemp seed cake (40% wheat flour substitution) [30]. In our study, the positive influence of hemp seed cake flour’s incorporation in bread formulation in terms of protein content was observed by the increase in the protein content from 11.11% d.m in the control sample to 18.18% d.m in HCB40%.

**Table 10:** Bread compositions and calculated nutritional values.

Bread Sample	Raw Materials	Amount, g/100 g Product	Energy, kcal	Protein, g/100 g	Carbohydrates, g/100 g	Fiber, g/100 g	Fat, g/100 g	Energy from Protein, %
HCB0%	WF, g	44.03	152.25	4.71	32.19	0.57	0.40	12.37
	HCF, g	0.00	0.00	0.00	0.00	0.00	0.00	
	L, g	24.77	32.04	0.99	6.78	0.12	0.08	
HCB5%	WF, g	42.03	145.38	4.50	30.72	0.55	0.38	13.32
	HCF, g	2.21	6.67	0.64	0.21	0.89	0.17	
	L, g	24.89	32.04	0.99	6.78	0.12	0.08	
HCB10%	WF, g	40.43	139.85	4.33	29.56	0.53	0.36	14.27
	HCF, g	4.49	13.54	1.30	0.42	1.80	0.34	
	L, g	25.27	32.04	0.99	6.78	0.12	0.08	
HCB15%	WF, g	38.34	132.60	4.10	28.02	0.50	0.35	15.59
	HCF, g	6.77	16.33	1.96	0.63	0.69	0.51	
	L, g	25.37	32.04	0.99	6.78	0.12	0.08	
HCB20%	WF, g	36.32	125.62	3.89	26.55	0.47	0.33	16.23
	HCF, g	9.08	27.37	2.63	0.85	3.65	0.68	
	L, g	25.54	32.04	0.99	6.78	0.12	0.08	
HCB30%	WF, g	31.99	110.64	3.42	23.38	0.42	0.29	18.23
	HCF, g	13.71	41.32	3.98	1.28	5.50	1.03	
	L, g	25.70	32.04	0.99	6.78	0.12	0.08	
HCB40%	WF, g	27.50	95.13	2.94	20.10	0.36	0.25	20.28
	HCF, g	18.33	55.26	5.32	1.72	7.36	1.38	
	L, g	25.78	32.04	0.99	6.78	0.12	0.08	

WF—wheat flour, HCF—hemp seed cake flour, L—leaven. HCB0%—control sample, HCB5%—bread with 5% addition of hemp seed cake flour, HCB10%—bread with 10% addition of hemp seed cake flour, HCB15%—bread with 15% addition of hemp seed cake flour, HCB20%—bread with 20% addition of hemp seed cake flour, HCB30%—bread with 30% addition of hemp seed cake flour, HCB40%—bread with 40% addition of hemp seed cake flour. Amounts of each material are presented next to the name of the material. Nutritional values correspond to the quantity each sample contains. The table only lists the ingredients which contain energy-providing nutrients. According to its label, the wheat flour composition was as follows: carbohydrates 73.1%, proteins 10.7%, fat 0.9%, fibre 1.3%. Total carbohydrates of HCF were calculated by difference [100 − (moisture + ash + protein + fat + fibre)]. Energy provided by dietary fibre (DF) fermentation was considered, equal to approximately 2 kcal according to the Food and Agriculture Organization of the United Nations [119].

The European Union’s Regulation No. 1924/2006 concerning nutrition and health claims made on foods states the following: “a claim that a food is a source of protein, and any claim likely to have the same meaning for the consumer, may only be made where at least 12% of the energy value of the food is provided by protein” and “a claim that a food is high in protein, and any claim likely to have the same meaning for the consumer, may only be made where at least 20% of the energy value of the food is provided by protein” [120]. According to this Regulation, HCB40% can be labelled as high in protein, because 20,28% of its energy value is provided by protein. On the other hand, according to the same document, all the bread samples can be labelled as sources of protein (Table 10). However, it has to be mentioned that the wheat protein is low in some essential amino acids; thus, wheat bread is not a complete protein source.

##### Colour Parameter Analysis

Bread colour was affected by the presence of hemp seed cake flour even when the last one was used in minimal amounts (5% reported to the wheat flour mass). The hemp seed cake flour itself was dark with a brown tint; therefore, the bread samples were darker (Table 11). As can be seen in Table 12, the values of the crust lightness (*L)* of hemp seed cake breads were always significantly (*p* ≤ 0.05) lower—up to 40% (for HCB40%) lower compared to control bread.

Crumb lightness was also significantly affected by wheat flour substitution with hemp seed cake flour. Incorporation of hemp seed cake flour led to a darker crumb, showing a decrease in lightness from 16.00% (HCB5%) to 54.93% (HCB40%) with respect to the control. Maravić et al. (2022) and Mohtarami (2019) explain this by the fact that when introducing a protein source in product formulations, more amino groups are available to be involved in Maillard reactions [121,122]. The highest colour difference (*ΔE*) in the crust as well as in the crumb was obtained for the breads with the highest amount of hemp seed cake flour. This fact is explained by the upward trend of chromatic parameter *a* (i.e., redness) simultaneously with the decreasing *b* (i.e., yellowness). Similar results were published by Miculek et al. (2019)—they associated an increase in *a* tint and decrease in *b* with hemp seed flour’s inclusion in breads [112]. All enriched bread samples were less white with lower values of the whiteness index.

##### Texture Analysis

Changes in the texture of new bread formulations were reported in several studies [123,124,125]. The study by Raczyk et al. (2022) shows changes in crumb texture properties of bread incorporated with tomato, beetroot and carrot juice. The observations of Guardianelli et al. (2023) show that with increases in the level of lupine flour, the chewiness and firmness of bread increased, while the elasticity decreased.

In our study, the addition of 5, 10 and 15% hemp seed cake flour, relative to the mass of wheat flour, produced a significantly softer bread texture (hardness showed a downward trend from 423.32 g to 102.21, 125.62 and 259.64, respectively) (Table 12). Further, with the addition of hemp seed cake flour at a level of 20 to 40%, the bread texture became harder than the wheat bread sample. It is known that hemp flour has a higher hydration level than wheat flour [126,127], and this can lead to softer bread. Thus, on one hand, at lower incorporation levels, the addition of hemp seed cake flour can lead to softer bread (HCB5%, HCB10% and HCB15%), and when supplementing bread formulations with 20, 30 and 40% hemp seed cake flour, the higher water absorption of hemp seed cake flour led to a denser dough and, consequently, a denser and harder bread.

A similar behaviour was noted in the case of cohesiveness, gumminess and chewiness, which, as explained by Nasir et al. (2020), is due to the hardening of the wheat flour, which results in crumbling as a consequence of the loss of water caused by the retrogradation properties of wheat flour [128]. It was also acclaimed that the changes in springiness and resilience were less pronounced.

Given the fact that hardness, gumminess and chewiness were textural parameters with the greatest changes with the incorporation of hemp flour, attempts were made to establish the effect of these textural parameters on overall acceptability. However, no significant correlation was detected; the correlation coefficients (*r*) were −0.29, −0.33 and −0.33, respectively. Likewise, no correlation was established between the texture parameters determined experimentally and the texture appreciated at the sensory level (Figure 2e). Meullenet et al. (1998) explained that the lack of this correlation is often caused by sensory data inaccuracy rather than by inappropriate selection of instrumental tests [129]. On the other hand, Gåmbaro et al. (2002) stated that sensory texture could be well predicted by instrumental texture measurements [130]. Both positive and negative correlations between sensorially and instrumentally determined bread textural indices were also obtained by Scheuer et al. (2016) [131]. However, it should be mentioned that in the studies of Gåmbaro et al. (2002) and Scheuer et al. (2016), only specially trained panellists in bread sensory analysis participated, while in our study, volunteers, regular bread consumers, participated.

In the industrial field, the use of hemp meal as an ingredient in the preparation of bread will contribute to alleviating the problem of waste management through valorisation, recovery, and recycling, which represents a national necessity and an economic and ecological priority. Thus, the production of bread with hemp meal will promote the sustainable economy, and it will favour cooperation between all economic branches to prevent ecological risks and the production of damage.

## 4. Conclusions

Hemp seed cake is a by-product resulting from oil extraction, and in most cases, it is used as animal feed. In spite of this, the chemical composition of this material and its low cost make it an attractive ingredient for the development of value-added food products. The analysis of the composition of hemp seed cake flour revealed high levels of phenolic compounds with strong antioxidant activity, the main phenolic compounds belonging to Cannabisin group. Hemp seed cake flour showed considerably high contents of essential amino acids, giving the proteins a quality that can ensure a harmonious growth of the body. According to the results, the incorporation of hemp seed cake flour in bread formulation positively contributes to the total phenol content, that increased from 141.25 mg GAE/kg (control sample) to 354.85 mg GAE/kg (40% hemp seed cake bread), and anti-radical activity. General bread quality parameters such as moisture content and water activity did not vary significantly. On the other hand, bread parameters like hardness, resilience and colour attributes were significantly affected by hemp seed cake flour addition. Bread sensory analysis revealed that HCB30% and HCB40% were less liked compared with the control, HCB5%, HCB10%, HCB15% and HCB20% samples; however, the overall appreciation of HCB30% and HCB40% was high enough (between “slightly like” and “like”) to state that this bread can find its consumer niche in the market. Our findings suggest that bread samples containing hemp seed cake flour at a level of 40% (*w*/*w* wheat flour) can be labelled as high in protein based on the corresponding EU regulation.

## Figures and Tables

**Table 1:** Bread formulations.

Raw Materials	Hemp Seed Cake Flour, % (% Replacement of Wheat Flour)						
	0	5	10	15	20	30	40
Wheat flour, g	1200	1140	1080	1020	960	840	720
Hemp seed cake flour, g	0	60	120	180	240	360	480
Water, mL	1100	1100	1100	1100	1100	1100	1100
Salt, g	25	25	25	25	25	25	25
Leaven, g	675	675	675	675	675	675	675

**Table 2:** CATA analysis terms.

Product Attribute	CATA Terms
Colour	too light, just the right colour, too dark, golden colour, grey colour
Odour	too strong hemp odour, too weak hemp odour, pine nut-like odour, woody odour, earthy odour, pleasant odour
Flavour	Tasty, sweet taste, fermented flavour, too strong flavour, too weak flavour
Texture	Hard, soft, crumbly, dry, moist, granular

**Table 3:** Physicochemical indicators of the wheat flour.

Indicator	Wheat Flour	Hemp (*Cannabis sativa* L.) Seed Cake Flour
Moisture content, %	14.10 ± 0.07 ^a^	8.24 ± 0.11 ^b^
Wet gluten content, %	24.80 ± 0.13	-
Ash content, % d.m.	0.49 ± 0.01 ^a^	6.21 ± 0.05 ^b^
Acidity, degrees	2.33 ± 0.06	-
Total protein content, % d.m	12.45 *	31.62 ± 0.22
Fat content, % d.m.	1.04 *	8.19 ± 0.13
Total dietary fibre content, % d.m.	1.51 *	43.76 ± 0.34

Results indicate the mean value of three independent assays and are expressed as mean ± standard deviation (SD); in each row, different letters ^a,b^ mean significant differences (*p* ≤ 0.05). * The values (%) were taken from the product label and converted into % d.m.

**Table 4:** Comparison of amino acid content of the hemp seed cake flour and wheat flour with protein pattern of FAO.

Amino Acid	FAO/WHO Standard (2013), mg/g [63]	Hemp Seed Cake Flour			Wheat Flour	
		Amino Acid Content, g/kg	g Amino Acid/100 g Protein	AAS, %	g Amino Acid/100 g Protein [85]	AAS, %
Threonine	2.3	15.95 ± 0.15	5.48 ± 0.13	238.00	2.7–3.1	126.08
Valine	3.9	8.42 ± 0.05	2.89 ± 0.11	74.10	4.3–4.8	116.66
Methionine	-	4.78 ± 0.07	1.64 ± 0.03		1.0–1.3	
Isoleucine	3.0	16.65 ± 0.24	5.72 ± 0.08	190.66	3.5–4.0	125.00
Leucine	5.9	23.69 ± 0.37	8.14 ± 0.07	137.97	6.7–7.1	116.94
Phenylalanine	-	18.17 ± 0.12	6.25 ± 0.02		4.1–4.9	
Lysine	4.5	11.23 ± 0.19	3.86 ± 0.08	85.77	2.5–3.2	63.33
Histidine	1.5	9.12 ± 0.11	3.14 ± 0.16	209.33	2.2–2.4	153.33
Methionine + Cysteine	2.2	5.96 ± 0.07	2.05 ± 0.14	93.18	3.1–4.1	163.63
Phenylalanine + Tyrosine	3.8	25.91 ± 0.13	8.91 ± 0.18	234.47	5.7–6.8	164.47
Σ am. essentials		119.30 ± 0.11	37.13 ± 0.24			
Arginine	-	31.81 ± 0.12	10.93 ± 0.08		4.1–4.7	
Σ am. semi-essential		31.81 ± 0.06	10.93 ± 0.17			
Aspartic acid	-	17.91 ± 0.22	6.16 ± 0.13		4.8–5.9	
Serine	-	20.47 ± 0.17	7.04 ± 0.11		4.5–4.7	
Glutamic Acid	-	60.26 ± 0.24	20.71 ± 0.12		29.0–32.8	
Proline	-	12.47 ± 0.14	4.29 ± 0.06		9.9–11.1	
Glycine	-	14.41 ± 0.16	4.95 ± 0.07		3.7–4.4	
Alanine	-	16.67 ± 0.07	5.73 ± 0.09		3.4–4.2	
Cysteine	-	1.18 ± 0.15	0.41 ± 0.04		2.1–2.8	
Tyrosine	-	7.74 ± 0.31	2.66 ± 0.08		1.6–1.9	
Σ am. non-essential		139.82 ± 0.45	51.94 ± 0.13			
γ-aminobutyric acid	-	0.95 ± 0.02	-		-	
Ammonia	-	3.23 ± 0.04	1.11 ± 0.04		3.1–3.8	
Σ free amino acids		291.88 ± 0.34	100.00 ± 0.17			
Σ am. proteinogenic		290.92 ± 0.28	100.00 ± 0.25			

Results indicate the mean value of three independent assays and are expressed as mean ± standard deviation (SD).

**Table 5:** Calculated protein efficiency ratio (PER) values.

Parameter	Value
PER1	2.83 ± 0.06
PER2	2.95 ± 0.03
PER3	3.40 ± 0.05

Results indicate the mean value of three independent assays and are expressed as mean ± standard deviation (SD).

**Table 6:** Mineral content of hemp (*Cannabis sativa* L.) seed cake flour.

Minerals	mg/100 g	PRI/AI, %
Mg	883.36 ± 5.86	252.39 ± 3.07
K	1594.95 ± 5.61	45.57 ± 1.15
P	1851.42 ± 4.73	336.62 ± 4.11
Mn	13.14 ± 0.07	438.38 ± 5.26
Fe	10.54 ± 0.09	95.82 ± 2.09
Zn	11.88 ± 0.14	84.85 ± 1.98
Cu	1.92 ± 0.06	120.12 ± 2.04

Results indicate the mean value of three independent assays and are expressed as mean ± standard deviation (SD).

**Table 7:** Identification and quantification of phenolic compounds in the hemp seed and seed cake flour samples, expressed in mg/kg.

Peak	R_t_ (min)	UV λ_max_ (nm)	[M + H]^+^ (*m*/*z*)	Phenolic Compound	Subclass	Relative Content, mg/kg	
						Seeds	Seed Cake Flour
1	2.81	270	123	Benzoic acid	Benzoic acid	19.40 ± 0.14 ^a^	32.61 ± 0.11 ^b^
2	2.94	270	139	2-Hydroxybenzoic acid	Hydroxybenzoic acid	35.46 ± 0.12 ^a^	59.60 ± 0.09 ^b^
3	9.47	280	155	Protocatechuic acid	Hydroxybenzoic acid	29.84 ± 0.10 ^a^	50.15 ± 0.12 ^b^
4	10.77	290	595	Cannabisin A	Lignanamide	10.57 ± 0.05 ^a^	17.76 ± 0.14 ^b^
5	11.41	290	597	Cannabisin B	Lignanamide	6.94 ± 0.07 ^a^	11.66 ± 0.09 ^b^
6	11.68	322, 290	301	*N-trans*-Caffeoyltyramine	Hydroxycinnamic acid amide	18.95 ± 0.11 ^a^	31.83 ± 0.07 ^b^
7	14.37	290	611	Cannabisin C	Lignanamide	9.02 ± 0.12 ^a^	15.16 ± 0.11 ^b^
8	14.66	290	597	Cannabisin B isomer	Lignanamide	4.73 ± 0.04 ^a^	7.94 ± 0.06 ^b^
9	16.22	320, 290	284	*N-trans*-Coumaroyltyramine	Hydroxycinnamic acid amide	33.51 ± 0.06 ^a^	56.31 ± 0.14 ^b^
10	18.07	322, 290	301	*N-trans*-Caffeoyltyramine isomer	Hydroxycinnamic acid amide	6.98 ± 0.08 ^a^	11.74 ± 0.03 ^b^
11	19.99	340, 290	597	Cannabisin Q	Lignanamide	3.04 ± 0.07 ^a^	5.11 ± 0.05 ^b^
12	20.25	330, 290	314	*N*-Feruloyltyramine	Hydroxycinnamic acid amide	4.37 ± 0.04 ^a^	7.35 ± 0.04 ^b^
13	22.97	290	625	Cannabisin F	Lignanamide	90.18 ± 0.19 ^a^	151.56 ± 0.28 ^b^
14	23.46	320, 290	625	Grossamide	Lignanamide	45.64 ± 0.15 ^a^	76.70 ± 0.16 ^b^
				Total phenols		318.62 ± 3.46 ^a^	535.49 ± 2.54 ^b^

Results indicate the mean value of three independent assays and are expressed as mean ± standard deviation (SD); in each row, different letters ^a,b^ mean significant differences (*p* ≤ 0.05).

**Table 8:** Bread samples viewed from above and cross-sections.

**Sample**	**HCB0%**	**HCB5%**	**HCB10%**
	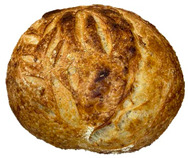	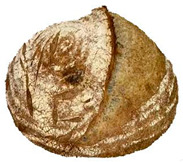	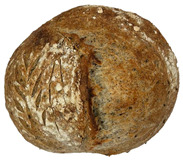
	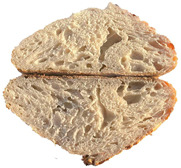	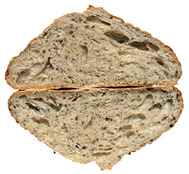	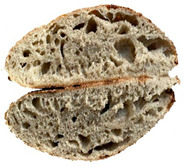
**HC 15%**	**HCB20%**	**HCB30%**	**HCB40%**
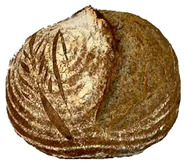	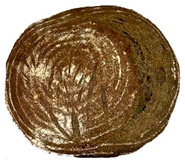	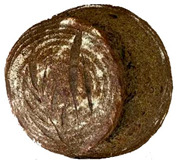	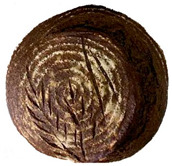
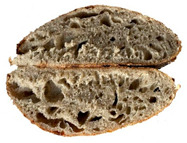	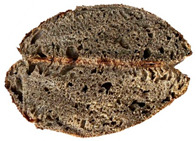	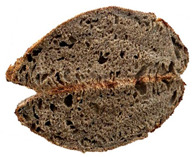	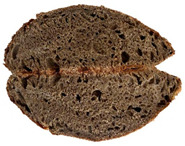

HCB0%—control sample, HCB5%—bread with 5% addition of hemp seed cake flour, HCB10%—bread with 10% addition of hemp seed cake flour, HCB15%—bread with 15% addition of hemp seed cake flour, HCB20%—bread with 20% addition of hemp seed cake flour, HCB30%—bread with 30% addition of hemp seed cake flour, HCB40%—bread with 40% addition of hemp seed cake flour.

**Table 9:** Quality parameters of bread samples.

Sample	HCB0%	HCB5%	HCB10%	HCB15%	HCB20%	HCB30%	HCB40%
Moisture content, %	45.76 ± 0.41 ^a^	45.43 ± 0.26 ^a^	47.21 ± 0.36 ^a^	46.52 ± 0.32 ^a^	46.69 ± 0.23 ^a^	47.07 ± 0.17 ^a^	47.27 ± 0.31 ^a^
Protein content, % d.m	11.11 ± 0.02 ^a^	11.83 ± 0.02 ^a^	13.16 ± 0.05 ^ab^	13.79 ± 0.07 ^ab^	14.70 ± 0.06 ^b^	16.47 ± 0.08 ^c^	18.18 ± 0.03 ^d^
Volume, cm^3^	2950.15 ± 13.85 ^a^	2255.85 ± 14.57 ^b^	2153.72 ± 16.31 ^c^	2106.65 ± 13.22 ^d^	2028.34 ± 15.61 ^e^	1935.81 ± 17.25 ^f^	1846.62 ± 14.24 ^g^
Baking loss, %	9.16 ± 0.04 ^a^	9.59 ± 0.07 ^a^	10.96 ± 0.05 ^a^	11.31 ± 0.08 ^ab^	11.89 ± 0.07 ^ab^	12.46 ± 0.06 ^b^	12.73 ± 0.02 ^b^
Porosity, %	74.45 ± 0.02 ^a^	73.15 ± 0.02 ^a^	71.89 ± 0.02 ^ab^	70.23 ± 0.02 ^b^	69.14 ± 0.02 ^b^	67.85 ± 0.02 ^c^	65.76 ± 0.02 ^d^
Acidity, degrees	1.55 ± 0.03 ^a^	1.63 ± 0.04 ^ab^	1.68 ± 0.03 ^ab^	1.72 ± 0.05 ^ab^	1.77 ± 0.06 ^b^	1.84 ± 0.03 ^bc^	1.91 ± 0.04 ^c^
Water activity (a_w_)	0.798 ± 0.005 ^a^	0.797 ± 0.006 ^a^	0.800 ± 0.007 ^a^	0.807 ± 0.003 ^a^	0.798 ± 0.005 ^a^	0.8000 ± 0.004 ^a^	0.798 ± 0.007 ^a^
Total Polyphenol Content, mg GAE/kg	148.25 ± 3.24 ^a^	182.35 ± 2.05 ^b^	216.17 ± 1.75 ^c^	225.84 ± 3.14 ^cd^	256,43 ± 4.42 ^d^	308.97 ± 3.78 ^e^	354.85 ± 2.87 ^f^
DPPH, mg Trolox/kg	228.25 ± 5.03 ^a^	318.58 ± 4.75 ^b^	377.85 ± 3.54 ^c^	393.86 ± 6.17 ^d^	452.47 ± 5.64 ^e^	534.18 ± 4.53 ^f^	627.55 ± 6.27 ^g^

Results indicate the mean value of three independent assays and are expressed as mean ± standard deviation (SD); in each row, different letters ^a–g^ mean significant differences (*p* ≤ 0.05). HCB0%—control sample, HCB5%—bread with 5% addition of hemp seed cake flour, HCB10%—bread with 10% addition of hemp seed cake flour, HCB15%—bread with 15% addition of hemp seed cake flour, HCB20%—bread with 20% addition of hemp seed cake flour, HCB30%—bread with 30% addition of hemp seed cake flour, HCB40%—bread with 40% addition of hemp seed cake flour.

**Table 11:** Colour parameters of bread samples with hemp seed cake flour.

	HCB0%	HCB5%	HCB10%	HCB15%	HCB20%	HCB30%	HCB40%
Crust colour							
L	58.55 ± 0.51 ^f^	46.00 ± 0.28 ^e^	44.80 ± 0.61 ^d^	42.69 ± 0.54 ^c^	39.17 ± 0.47 ^b^	38.93 ± 0.53 ^b^	34.79 ± 0.69 ^a^
a	4.77 ± 0.02 ^a^	5.74 ± 0.03 ^b^	7.66 ± 0.10 ^c^	7.70 ± 0.08 ^cd^	7.96 ± 0.08 ^d^	8.11 ± 0.08 ^e^	8.86 ± 0.07 ^f^
b	11.21 ± 0.08 ^a^	15.78 ± 0.11 ^b^	19.17 ± 0.16 ^c^	19.22 ± 0.05 ^c^	19.73 ± 0.09 ^cd^	20.23 ± 0.11 ^d^	21.88 ± 0.25 ^e^
ΔE		13.39 ± 0.09 ^a^	16.15 ± 0.13 ^b^	18.01 ± 0.06 ^c^	21.41 ± 0.15 ^d^	21.85 ± 0.07 ^e^	26.37 ± 0.14 ^f^
Crumb colour							
L	60.84 ± 0.54 ^f^	51.11 ± 0.68 ^e^	46.37 ± 0.75 ^d^	45.82 ± 0.64 ^cd^	44.63 ± 0.38 ^c^	37.95 ± 0.56 ^b^	27.42 ± 0.26 ^a^
a	−0.05 ± 0.01 ^a^	−0.57 ± 0.01 ^b^	0.15 ± 0.02 ^c^	0.25 ± 0.01 ^d^	0.66 ± 0.01 ^e^	0.96 ± 0.01 ^f^	1.35 ± 0.02 ^g^
b	13.47 ± 0.09 ^f^	13.16 ± 0.08 ^e^	12.51 ± 0.14 ^d^	12.07 ± 0.09 ^c^	11.67 ± 0.07 ^cb^	11.00 ± 0.09 ^b^	8.81 ± 0.11 ^a^
ΔE		9.75 ± 0.06 ^a^	14.50 ± 0.07 ^b^	14.54 ± 0.11 ^b^	16.33 ± 0.13 ^c^	23.05 ± 0.15 ^d^	33.77 ± 0.34 ^e^
WI	58.59 ± 0.56 ^f^	49.37 ± 0.39 ^e^	44.93 ± 0.43 ^d^	44.49 ± 0.31 ^cd^	43.41 ± 0.63 ^c^	36.98 ± 0.52 ^b^	26.87 ± 0.46 ^a^

Results indicate the mean value of three independent assays and are expressed as mean ± standard deviation (SD); in each row, different letters ^a–g^ mean significant differences (*p* ≤ 0.05). HCB0%—control sample, HCB5%—bread with 5% addition of hemp seed cake flour, HCB10%—bread with 10% addition of hemp seed cake flour, HCB15%—bread with 15% addition of hemp seed cake flour, HCB20%—bread with 20% addition of hemp seed cake flour, HCB30%—bread with 30% addition of hemp seed cake flour, HCB40%—bread with 40% addition of hemp seed cake flour. *L*—colour lightness, a—redness/greenness, *b*—yellowness/blueness, *WI*—whiteness index.

**Table 12:** Texture profile analysis (TPA) of bread supplemented with hemp seed cake flour.

	Hardness, g	Springiness, %	Cohesiveness, %	Resilience, %	Gumminess, g	Chewiness, g
HCB0%	423.32 ± 12.58 ^d^	1.001 ± 0.011 ^a^	0.885 ± 0.005 ^a^	0.638 ± 0.002 ^b^	374.25 ± 14.2 ^d^	374.43 ± 15.62 ^d^
HCB5%	102.21 ± 4.21 ^a^	1.001 ± 0.009 ^a^	0.923 ± 0.005 ^a^	0.617 ± 0.003 ^ab^	94.57 ± 3.98 ^a^	94.62 ± 3.98 ^a^
HCB10%	125.62 ± 5.98 ^b^	1.001 ± 0.007 ^a^	0.904 ± 0.002 ^a^	0.630 ± 0.005 ^b^	113.34 ± 4.58 ^b^	113.39 ± 5.21 ^b^
HCB15%	259.64 ± 8.85 ^c^	1.001 ± 0.007 ^a^	0.843 ± 0.003 ^a^	0.583 ± 0.004 ^a^	218.97 ± 12.35 ^c^	219.08 ± 7.85 ^c^
HCB20%	530.37 ± 14.48 ^e^	1.001 ± 0.009 ^a^	0.863 ± 0.006 ^a^	0.616 ± 0.001 ^ab^	540.73 ± 14.84 ^f^	541.00 ± 21.05 ^f^
HCB30%	626.66 ± 24.87 ^f^	1.001 ± 0.008 ^a^	0.836 ± 0.003 ^a^	0.569 ± 0.005 ^a^	443.91 ± 18.96 ^e^	444.14 ± 17.62 ^e^
HCB40%	642.88 ± 26.65 ^g^	1.001 ± 0.007 ^a^	0.847 ± 0.002 ^a^	0.562 ± 0.005 ^a^	544.41 ± 21.25 ^f^	544.68 ± 18.54 ^f^

Results indicate the mean value of three independent assays and are expressed as mean ± standard deviation (SD); in each column, different letters ^a–g^ mean significant differences (*p* ≤ 0.05). HCB0%—control sample, HCB5%—bread with 5% addition of hemp seed cake flour, HCB10%—bread with 10% addition of hemp seed cake flour, HCB15%—bread with 15% addition of hemp seed cake flour, HCB20%—bread with 20% addition of hemp seed cake flour, HCB30%—bread with 30% addition of hemp seed cake flour, HCB40%—bread with 40% addition of hemp seed cake flour.

## Data Availability

The data presented in this study are available on request from the corresponding author.
